# Recombination map construction method using ONT sequence

**DOI:** 10.1016/j.mex.2022.101969

**Published:** 2022-12-16

**Authors:** Zuoquan Chen, Lei Xie, Xi Tang, Zhiyan Zhang

**Affiliations:** State Key Laboratory for Pig Genetic Improvement and Production Technology, Jiangxi Agricultural University, Nanchang 330045, China

**Keywords:** Recombination, ONT sequences, Pipelines, Computational biology, Recombination map construction method using ONT sequence

## Abstract

Although meiotic recombination is a key step shared by eukaryotes, the rate of recombination varies at different taxonomic levels. The construction of high-resolution genome-wide recombination maps will help us understand the variability patterns of recombination rates and their molecular basis. ONT sequencing technology has the characteristics of long read length, high throughput, and reasonable cost, and can be used as a data source for the construction of whole-gene recombination landscapes. In order to construct the genome-wide recombination map of an individual conveniently and accurately, we developed a method to construct the recombination landscape based on the third-generation sequencing technology, Oxford Nanopore Sequencing. Here we detail a step-by-step approach to efficiently and accurately construct genome-wide recombination maps using ONT pooled sequencing data. The main contents include compression homopolymers and alignment; acquisition of high-quality variants; estimation of recombinant molecules by the sliding window method; and construction of recombinant maps. The results of simulation data validation show that our method has high sensitivity and specificity at moderate heterozygous variant density and sequencing depth. This method provides a new way of constructing high-resolution individual genome recombination maps using long read sequences, and has important reference significance for the study of recombination rate variation.

## Highlights

• For the first time, a detailed method for constructing an individualized genome-wide recombination map using ONT pooled sequencing data has been introduced.
• Our method performs well in estimating recombinant molecules with moderate heterozygous variant densities and sequencing depths.
• The method's detailed pipeline code is simple to use and download: https://github.com/zuoquanchen/NanoCross

## Graphical abstract

**Figure:**
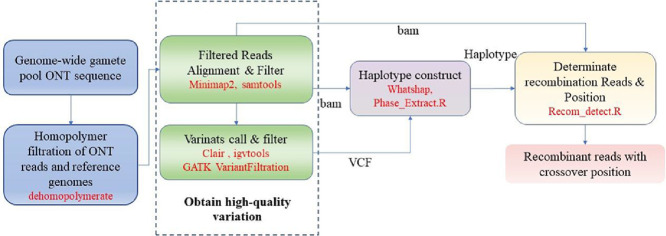

Specifications table

**Table:** 

Subject area:	Bioinformatics
More specific subject area:	genomics
Name of your method:	Recombination map construction method using ONT sequence
Name and reference of original method:	NanoCross: A pipeline that detecting recombinant crossover using ONT sequencing data
Resource availability:	The source code and data are stored in GitHub (https://github.com/zuoquanchen/NanoCross)

## Introduction

It is commonly known that recombination is the foundation of meiosis in eukaryotes and that meiotic recombination permits the correct separation of homologous chromosomes, thereby allowing offspring to generate new phenotypes. Meiotic recombination contributes significantly to genetic diversity and species evolution [1]. Recombination is central to genetic analysis, and although recombination maps were originally constructed primarily to aid in the generation of physical maps, they are now regarded as an essential tool for studying genome biology [2].

Currently, estimates of recombination rates are primarily derived from large microarray population data and pedigree information, which require a significant investment of time and resources [3]. Some researchers have proposed using gamete pool sequencing to construct individual genome-wide recombination maps in an efficient and cost-effective manner. Due to the fact that linked-reads have a long sequence span, low sequencing cost, and high sequencing accuracy, some studies have constructed recombination landscapes using next-generation sequencing-based Linked-reads gamete pool sequencing [4,5] but not third-generation sequencing (ONT sequencing, PacBio sequencing).

In 2012, Oxford Nanopore Sequencing Technology Co., Ltd. introduced Nanopore sequencing technology, a long-read sequencing technology [6]. It features single-molecule sequencing, real-time monitoring of sequencing data, ultra-long read lengths, and an affordable price. In some fields (genome research, microbial identification, disease medical research, etc.), the use of ONT sequencing technology is on the rise, despite the fact that second-generation short-read sequencing technology continues to hold a dominant position in the global sequencing market [7]. The disadvantage of nanopore sequencing is the high error rate. In many cases it is necessary to increase the coverage (which increases the cost of sequencing) or to complement with Illumina technology to reduce or correct the errors. Previous research has demonstrated that homopolymer compression can reduce machine-induced sequencing errors without altering the alignment of filtered reads to the compressed reference genome [8]. This paper presents a novel method that combines the benefits of homopolymer filtering and ONT sequencing to identify cross-recombination throughout the genome by rebuilding ONT molecular haplotype information.

This is the first study to suggest a way to build an individual genome-wide recombination map using the long-read sequencing technology ONT sequencing. This method combines the benefits of homopolymer filtering and ONT sequencing to accurately find crossover recombination across the whole genome by restoring ONT molecular haplotype information.

## Method details

Through ONT gamete pooling sequencing, homopolymer compression alignment, high-quality variant detection, haplotype construction, and recombinant molecular recognition, this research aims to construct individual genome-wide recombination maps quickly, affordably, and precisely using a large number of spermatozoa from a single individual. To specifically achieve this objective, our strategy is comprised of the following components:

### Obtain gamete samples for DNA extraction

Collect samples of gametes (Since egg samples are difficult to obtain, gamete generally refers to sperm samples). The preparation of sperm samples followed the protocol of Avery Davis Bell et al. High molecular weight genomic DNA was prepared by the CTAB method and followed by purification with QIAGEN® Genomic kit (Cat#13343, QIAGEN) for regular sequencing, according to the standard operating procedure provided by the manufacturer. The DNA degradation and contamination of the extracted DNA was monitored on 1% agarose gels. DNA purity was then detected using NanoDrop ™ One UV-Vis spectrophotometer (Thermo Fisher Scientific, USA), of which OD260/280 ranging from 1.8 to 2.0 and OD 260/230 is between 2.0-2.2. At last, DNA concentration was further measured by Qubit® 4.0 Fluorometer (Invitrogen, USA).

### ONT library building and sequencing

After the DNA quality inspection of gamete samples was qualified, the large fragments were recovered by cutting gel using BluePippin automatic nucleic acid recovery instrument (Sage Science, USA); DNA was damaged and repaired; after purification, the ends of DNA fragments were repaired and added with A After purification, the ligation reaction was performed using the adapters in the LSK109 ligation kit (Cat#SQK-LSK109, Oxford), and finally the constructed DNA library was accurately quantified with a Qubit® 3.0 Fluorometer (Invitrogen, USA). After the library was constructed, a certain concentration and volume of DNA library was added to one Flow cell, and the Flow cell was transferred to Nanopore GridION X5/PromethION (Oxford Nanopore Technologies, UK) for real-time single-molecule sequencing. Determine the amount of off-machine data of the ONT sequencer based on the size of the reference genome of the experimental animal and the required sequencing depth (generally the sequencing depth should reach about 60X).

### Raw reads and reference genome sequence homopolymer filtration

Because ONT sequencing data has a higher sequencing error rate than next-generation sequencing, especially when consecutive identical nucleotides are prone to more sequencing errors through nanopores. We compressed identical nucleotides in the reads using a homopolymer filtering operation to lower the sequencing error rate [9] (for example, Y=AGTTTCG, Y'=AGTCG after compression). Specifically, we use the homopolymer compression tool dehomopolymerate (https://github.com/tseemann/dehomopolymerate) developed by Torsten Seemann. To prevent subsequent alignment mismatches, we also needed to perform homopolymer filtering on the reference genome sequence as well. Homopolymer filtering can not only reduce the sequencing error rate to a certain extent but also reduce the data size and the consumption of computing resources.

### Sequence alignment and variant calling

In this step, the ONT reads after homopolymer compression should be aligned to the reference genome after homopolymer compression. Due to the fact that alignment results frequently contain multiple alignments and alignments with poor quality, we used samtools [10] to remove multiple alignments from the alignment files and sequences with an alignment quality of less than 60 in order to reduce their impact on subsequent variant calls. Then, using the filtered bam file and the reference genome as input files, the fast and accurate variant calling software Clair [11] (‘callVarBam’ command) is used to call variants. Select the highest quality cutoff (the valley between two peaks plus 50). Then, using the ‘VariantFiltration’ function of the GATK [12], we filtered variants whose variant quality was below the optimal quality threshold. In order to reduce interference, we also filtered clustered variants containing more than three variants within 10 bases to obtain variants of high quality.

### Haplotype construction

After obtaining high-quality variants, we used the phase option of Whatshap software [13] to reconstruct individual haplotypes using the high-quality variants, filtered bam files, and homopolymer-compressed reference genome instruments as input. To improve the accuracy of haplotype construction, structural variants such as deletions, duplications, copy number variations, and translocations, which can cause reads to be misaligned and incorrectly called, are disregarded. Also, since the number of sequence coverage loads affects how long the Whatshap software takes to run and how well it works, we used a maximum coverage parameter of 20 to quickly and accurately reconstruct individual haplotypes.

### Sliding window method for recombinant molecular estimation

To retrieve recombinant molecules from sperm pool sequencing sequences, we developed a sliding-window phasing ONT molecular orientation method that uses BAM files and haplotype files as inputs. To determine whether the reads are recombinant molecules, the method first estimates the haplotype score vector of the reads and then estimates the phase of the reads based on the haplotype score vector. We implemented the method in R3.4.3 [14], used the R package “Rsamtools” [15] to load the bam files, and parallelized this process using the R packages "foreach" and "dopar" to improve computational efficency. Specifically, each read undergoes the following processes:

a) Extract the CIGAR value of reads, and using the alignment position and CIGAR value, calculate the genome range spanned by the reads. According to the intersection of the genome range spanned by the reads and the positions in the haplotype file, generated genotype vectors for the positions corresponding to the reads.
b) According to the following formula, the read haplotype score vector is generated by comparing the genotype and haplotype information at the same position.
  (1) $$H_{k}=\left\{ \begin{array}{c} \;1,\;if\;G_{n}=H\_ref_{n}\;\\ -1,\;if\;G_{n}=H\_alt_{n}\\ \;0,\;Else\; \end{array}\right.$$
c) Traverse the haplotype score vector from the fourth position to the fourth from last position. A window contains position n as well as seven positions from the three positions before and after it, with a sliding step of one. Our method permits the existence of unknown haplotypes due to insertions, deletions, and sequencing errors. But each haplotype must be represented by at least 5 variants within a window. If not, the locus is said to be phase-deleted (NA), and the phase of the n position is estimated as follows:
  (2) $$\left\{ \begin{array}{c} Hap\;A,\;if\;\frac{\sum_{n-3}^{n+3}H_{k}}{L_{n}}\;\geq \;\frac{4}{6}\\ Hap\;B,\;if\;\frac{\sum_{n-3}^{n+3}H_{k}}{L_{n}}\;\leq \;-\frac{4}{6}\\ NA,\;Else\; \end{array}\right.$$
  where $L_{n}$ is the number of loci in the n-window with a non-zero haplotype score.
d) Finally, the phase vector after the sliding window is then used to determine whether the read has phase switching. It is considered a recombination molecule if it exists.

### Filter recombinant molecules in covering abnormal regions

The estimation of recombinant molecules relies on correct haplotype formation, which has a high correlation with sequencing depth. To reduce miscalculation of recombinant molecules caused by anomalous alignment coverage, we computed the coverage of bam files using the depth command of the samtools software. The in-position sequencing depth of the recombined molecules is used to figure out the average sequencing depth. Recombination molecules with abnormal average coverage (average depth) will not be used to make the recombination map.

### Create the reference and compressed genomes' location correspondence file

Since our recombination molecules were estimated on the basis of homopolymer-compressed reference genomes, the original recombination locations were not directly known when constructing the recombination landscape. In order to obtain the original recombination position interval of the recombinant molecule, we used the "Biostrings" package in R to read the pig reference genome, traverse the sequence by chromosome, construct the position correspondence between the chromosomes of the reference genome and the chromosomes of the compressed genome according to the principle of homopolymer compression, and finally get the location corresponding files of the reference genome and the compressed genome. According to the position correspondence file, we can restore the recombination position interval of the recombination molecule estimated above to the original position.

### Construction genome-wide recombination landscape

After determining the number of recombinant molecules, we created recombination graphs in 1 Mb windows using the ggplot2 [16] tool in R. Specifically, the table function is used for different chromosomes to count the number of recombined molecules across groups based on a 1 Mb window, and a histogram is generated for the number between groups in order to obtain the recombination landscape of the entire genome. In particular, because the homologous pairing of sex chromosomes (X and Y chromosomes) occurs only in the pseudoautosomal area, the recombination molecular estimation and the building of the whole genome recombination map do not take into account the sex chromosomes.

### Method validation

**Fig. 1:**
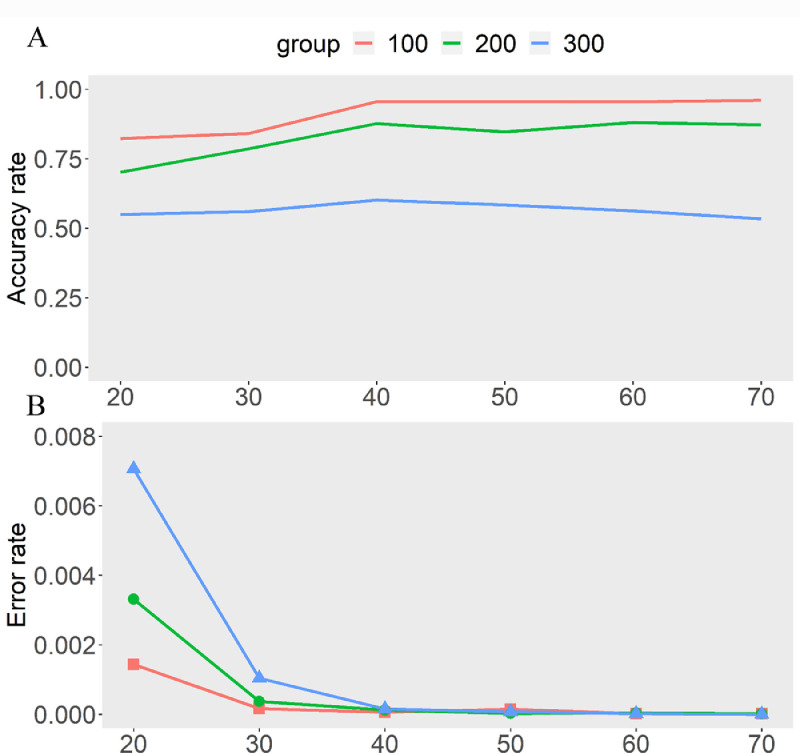
Accuracy and error rates for simulated dataset tests. The X-axis represents the different sequencing depths, and the Y-axis is the estimated error rate (bottom panel) and accuracy rate(top panel), respectively. Different colors represent different mean heterozygous variation densities.

To verify the accuracy and performance of our method, we used an ONT read simulator NanoSim [17] to simulate different reconstituted datasets. The first step of NanoSim is read characterization, which provides a comprehensive alignment-based analysis and generates a set of read profiles serving as the input to the next step, the simulation stage. We used the different haplotypes and a set of read profiles generated in the first step as input to the second simulation stage. With different haplotype reference inputs, NanoSim can generate two ONT reads datasets without recombinant molecules and one ONT reads dataset with recombinant molecules. Three different datasets were combined to simulate a gamete pool with a mixture of recombinant and non-recombinant molecules. Genome heterozygous variation density and sequencing coverage are generally considered to be important factors affecting the correctness of recombinant molecule detection, so we simulated 3 data sets in total. Different data sets have different heterozygous variation densities, and each group Contains 6 subsets with different total reads to simulate different average sequencing coverage. We calculated the accuracy rate (sensitivity) as the proportion of NanoCross correctly identifying recombinant molecules in all simulated recombinant molecules and then calculated the error rate as the proportion of misidentified recombinant molecules in all non-recombinant molecules. The results showed that the accuracy rate is above 90% at an average of 100bp per SNV. The detection accuracy and specificity were significantly influenced by the average sequencing—the higher of sequencing depth, the more accuracy of reads crossover detection. The highest error rate was (0.0070625) at an average sequencing depth of 20 and an average variant density of 300bp per SNV (Fig. 1, Table1). In addition, we applied our method to ONT sequencing data of a wild boar sperm pool in a previous study, and the results showed that our method can obtain high-resolution recombination maps more accurately [18]. Overall, our technique performs admirably with moderate heterozygous variation and sequencing depth.

**Table 1:** Results table for simulation validation.

		Case^b^			Control		
Depths	Recombination^a^	100	200	300	100	200	300
20X	1	0.823	0.702	0.550	2.3	5.3	11.3
30X	2	1.682	1.572	1.120	0.4	0.9	2.5
40X	3	2.866	2.630	1.805	0.2	0.4	0.5
50X	4	3.820	3.388	2.336	0.6	0.1	0.3
60X	5	4.774	4.402	2.813	0.1	0.2	0.1
70X	6	5.758	5.231	3.204	0.1	0.1	0

a Represents the number of simulated recombination events in the data set.

b A simulated data set containing the recombination event.

## CRediT authorship contribution statement

**Zuoquan Chen:** Methodology, Software, Validation, Data curation, Writing – original draft, Writing – review & editing. **Lei Xie:** Methodology, Validation. **Xi Tang:** Data curation, Methodology. **Zhiyan Zhang:** Methodology, Software, Validation, Data curation, Writing – review & editing, Supervision.

## Declaration of Competing Interest

The authors declare that they have no known competing financial interests or personal relationships that could have appeared to influence the work reported in this paper.

## Data Availability

Data will be made available on request.
